# Septic arthritis and osteomyelitis of the pubic symphysis – a retrospective study of 26 patients

**DOI:** 10.5194/jbji-7-35-2022

**Published:** 2022-02-25

**Authors:** Rehne Lessmann Hansen, Mats Bue, Anna Bertoli Borgognoni, Klaus Kjær Petersen

**Affiliations:** 1 Department of Orthopaedic Surgery, Aarhus University Hospital Palle Juul-Jensens Blvd. 99, 8200 Aarhus, Denmark; 2 Department of Clinical Medicine, Aarhus University Palle Juul-Jensens Blvd. 99, 8200 Aarhus, Denmark

## Abstract

**Introduction**: Septic arthritis and osteomyelitis of the pubic symphysis (SAS) are rare conditions with nonspecific symptoms leading to diagnostic delay and treatment. **Aim**: We draw awareness to this condition elucidating the diagnostic procedures, surgical intervention and antibiotic management. **Methods**: This entail a retrospective follow-up study of 26 consecutive patients, median age of 71 years (range: 48–89) surgically treated for septic arthritis of the pubic symphysis between 2009 and 2020. Patient files, diagnostic imaging and bacterial cultures were evaluated. **Results**: Before diagnosed with SAS, 21 of the patients had previous pelvic surgery (16 due to malign conditions, 5 due to benign conditions), while 5 of the patients were not previously operated. Median follow-up period after SAS surgery was 18.5 months (range: 8 to 144.5 months). Dominating symptoms were severe suprapubic/pubic pain ( n = 26), gait difficulties ( n = 10) and intermittent fever ( n = 9). Diagnostic delay was between 1 and 12 months. The diagnostic imaging included magnetic resonance imaging (MRI) ( n = 24), computer tomography (CT) ( n = 17) and/or PET-CT ( n = 10), predominantly displaying bone destruction/erosion of the symphysis ( n = 13), abscess ( n = 12) and/or fistula ( n = 5) in the adjacent muscles. All patients underwent surgical debridement with resection of the symphysis and received a minimum of 6 weeks antibiotic treatment. Fourteen patients presented with monocultures and 4 patients with polycultures. Five patients underwent at least one revision surgery. Twenty-three patients experienced postoperative pain relief at 6 weeks follow-up, and 19 patients were ambulant without walking aids. **Conclusion**: SAS are rare conditions and should be suspected in patients with infection, pubic pain and impaired gait, especially after pelvic surgery. Bone infection, abscess and fistula near the symphysis can be visualized with proper imaging, most frequently with MRI. For most patients in this cohort surgical debridement combined with a minimum of 6 weeks antibiotic treatment resulted in pain relief, improved walking ability and a low recurrence rate.

## 1 Introduction

Septic arthritis and osteomyelitis of the pubic symphysis (SAS) are rare conditions, representing less than one percent of all cases of osteomyelitis cases (el Mezouar et al., 2014), and affect different groups of patients by several distinct aetiologies (Dudareva et al., 2017). Patients typically present with multiple comorbidities and with long-term pubic pain as the major complaint (Dudareva et al., 2017). The sources of infection are most often intra-abdominal or pelvic, e.g., following gastrointestinal, gynaecological, or urological surgery, or osteoradionecrosis following pelvic radiotherapy. The infection causes osseous destruction, edema and/or abscesses in the pubic symphysis and surrounding muscles. The resulting distinct pubic pain is often ascribed as post-operative related or associated with previous radiotherapy. Some mistake the symptoms as osteitis pubis, a noninfectious inflammatory condition affecting the pubic symphysis, previously described in patients undergoing urologic procedures, following trauma, in pregnancies and in athletes with groin pain (Gomella and Mufarrij, 2017). The diagnosis of SAS is commonly missed or delayed due to the infrequency of the disease and its variable presentation, resulting in patients repeatedly being bounced between specialties before correct diagnosis (Alaya et al., 2017). This may lead to an unintended subtle progression of the infection making the treatment more difficult. Due to its rare presentation, previous studies on SAS patients have predominantly been case reports focusing on the outcome after antibiotic treatment or surgical treatment.

This single-center retrospective study reports a series of 26 consecutive patients surgically treated for SAS. With this study, we aim to draw attention to these rare conditions, elucidating and discussing risk factors, diagnosis, bacteriology, and surgical and antibiotic management.

## 2 Methods

### 2.1 Study settings and participants

Twenty-six consecutive patients surgically treated at the Department of Orthopaedic Surgery, Aarhus University Hospital, between 2009 and 2020 were included.

SAS was defined as having pain in the pubic area, radiographic changes in the pubic region, as well as positive cultures or pus when debrided. Diagnostic delay was defined as the time from onset of clinical symptoms documented in the patient files and/or radiographic changes until SAS was diagnosed.

Patient files, radiology and microbiological cultures were retrieved from medical paper reports (2009 to 2012) and digital medical reports (Systematic columna, version 32.1) (2013 to 2021). Through the digital medical platform it was possible to identify patient admittances to other hospitals and read the patient records to identify relapse of infection. Patient records were screened for recurrence until 1 March 2021 or death. Data were handled according to the regulations of the Danish Data Protection Agency and approved by Central Denmark Region (registration number 1-45-70-14-20).

### 2.2 Surgical management

A bladder catheter was inserted to empty the bladder and locate the urethra. In supine position, the pubic symphysis was accessed through a Pfannenstiel incision or a lower laparotomy depending on the cicatrice from former surgery. The symphysis anatomy identification was typically difficult due to pus, granulation tissue, and breakdown of the fibrocartilaginous disc and the ligaments (including the arcuate ligament). Blunt Hohmann retractors were placed on the anterior and posterior side of the symphysis, followed by joint and infected juxta-articular bone removal with Luer forceps. The pubic bone, rami superior and inferior were debrided with curettage of all necrotic and infectious bone. Abscesses were surgically drained intraoperative or perioperative with ultrasound guided drainage. Careful hemostasis in the retropubic area (fossa Retzius) was performed. Due to early cases with postoperative hematoma, a 14 d surgical drain was routinely placed in the retropubic area and removed after 2–3 d. The area was washed with isotonic saline solution, and 1–2 representative tissue samples from the infected area were collected using a sterile technique. A 10×10 cm GENTA-COLL sponge containing 200 mg gentamicin sulfate was placed in the debrided area, and intravenous antibiotic 1500 mg cefuroxime was administered. One surgeon (Klaus Kjær Petersen) performed all the surgeries, but most surgical procedures were joint ventures with surgeons from relevant specialties.

**Figure 1:**
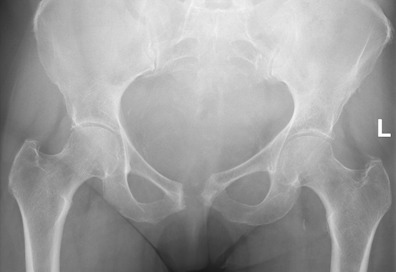
Patient with wide symphysis resection after SAS debridement. At the 6 weeks follow-up, the patient was able to stand on one leg and walk with two crutches. The examination was performed at the Department of Radiology, Aarhus University Hospital, Denmark.

Postoperative aftercare included patient immobilization for two days to avoid bleeding in the debrided area. Later the patients were mobilized with crutches or with a walking frame. Patients who underwent a wide resection of the symphysis (Fig. 1) and patients with former radiotherapy were susceptible to pelvic insufficiency fracture and had a restricted rehabilitation program with reduced weight-bearing the first 6 weeks.

### 2.3 Antibiotic treatment 

Antibiotics were paused ≥ 7 d before surgery unless continuous administration was needed due to bacteremia.

After surgical debridement, all patients were administered empiric intravenous cefuroxime 1500 mg three times a day until microbiological culture report, and hereafter antibiotic treatment was guided according to the antibiotic sensitivity. The patients received intravenous antibiotics for a total of 2 weeks and oral antibiotics for 4 weeks. If the culture report was negative but intraoperative pus present, empiric antibiotic continued for 6 weeks. In collaboration with a microbiology specialist, the duration of the antibiotic treatment was prolonged if the patient had other infection foci or slow clinical/biochemical response.

### 2.4 Statistics

Data were collected in an Excel spreadsheet (Microsoft, Redmond, Washington) and analyzed using the Excel software. All data were considered to be non-parametric and reported as median values and range.

## 3 Results 

### 3.1 Patient characteristics

The cohort comprised 10 females and 16 males with a median age of 70.5 years (range: 48 to 89) at the time of the primary surgery. Symptoms were severe suprapubic/pubic pain ( n = 26), gait difficulties ( n = 10), and intermittent fever ( n = 9), and one patient had a sinus tract. Diagnostic delay was between 1 and 12 months. Patient characteristics are presented in Table 1. Four patients died during this study period. No patients died of SAS. Patients were clinically evaluated in the orthopedic outpatient clinic median 1.5 (range: 0.5 to 7) months after final surgery. Median follow-up period was 18.5 months (range: 8 to 144.5 months).

**Table 1:** Patient characteristics, imaging and microbiological results.

ID	Referring	Predisposing	Surgery	Radio-	Diagnostic	Imaging	Imaging	Microorganism	Antibiotic	Revision
	department	conditions		therapy	delay (months)		results			surgery
1	urology	stress incontinence	mesh surgery	missing	12	CT + MRI	missing	*Escherichia coli*	unknown	revised
2	gynaecology	vulva cancer	vulvectomy	missing	1.5	CT	bone	Fusobacteria	unknown	
3	medicine	*Colitis ulcerosa*	no	missing	12	MRI + PET/CT	missing	*Staphylococcus aureus*	unknown	
4	urology	prostate hypertrophy	TURP surgery, penile implant	missing	8	MRI + PET/CT	missing	none	unknown	revised
5	gynaecology	vulva cancer	cancer surgery	missing	3	MRI + PET/CT	bone + abscess	*Bacteroides*	unknown	
6	urology	stress incontinence	bladder reconstruction	no		MRI	joint + marrow + fistula	none	unknown	
7	orthopedic	prostate cancer	none	yes	4	CT + MRI	bone + joint + marrow + abscess	coryneform bacteria,coagulase-negative staphylococci	yes	revised
8	urology	cervix cancer	hysterectomy	yes	2	CT + MRI + PET/CT	bone + joint + marrow + abscess	*Enterococcus faecalis*	yes ${}^{*}$	
9	urology	bladder cancer	cystectomy, Bricker bladder	yes	2	MRI	bone + abscess	none	yes ${}^{*}$	revised
10	urology	prostate cancer	TURP surgery	yes	3	CT	fistula	*Enterococcus faecalis*	yes ${}^{*}$	
11	medicine	none	none	no	2	CT + MRI	bone + joint + marrow + abscess	*Salmonella* species	paused	
12	urology	prostate cancer	prostectomy	no	6	MRI + PET/CT	bone + joint + increased FDG	none	paused	
13	urology	prostate cancer	prostectomy	yes	2	CT + MRI + PET/CT	bone + joint + marrow + sinus + increased FDG	*Streptococcus* (polyculture), *Escherichia coli*	paused	revised
14	infection	none	none	no	missing	CT + MRI + PET/CT	joint + marrow + abscess + increased FDG	*Staphylococcus aureus*	yes ${}^{*}$	
15	orthopedic	prostate hypertrophy	TURP surgery, recent pelvic fracture	no	1	CT + MRI	joint + marrow + abscess	*Staphylococcus aureus*,coagulase-negativestaphylococci	yes ${}^{*}$	
16	urology	prostate cancer	cryotherapy, Bricker bladder	yes	2	CT + MRI	bone + joint + marrow	*Enterococcus faecalis*,coagulase-negative staphylococci, *Bacteroidesfragilis*, *Klebsiella pneumoniae*	yes ${}^{*}$	
17	urology	prostate cancer	prostectomy	yes	4	CT + MRI	bone + marrow + fistula	*Pseudomonas aeruginosa*	yes	
18	urology	prostate cancer	TURP surgery	yes	6	CT + MRI + PET/CT	joint + marrow + fistula + increased FDG	none	paused	
19	gastroenterology	anal cancer	radical anal surgery	yes	3	CT + MRI	joint + marrow + abscess	*Streptococcus*	paused	
20	urology	prostate cancer	prostectomy	yes	1	MRI	joint + marrow	*Staphylococcus aureus*	paused	
21	urology	prostate cancer	prostectomy	yes	2	MRI	bone + joint + marrow	*Enterococcus faecalis*	paused	
22	urology	prostate cancer	prostectomy	yes	3	CT + MRI	joint + marrow + fistula	*Enterococcus faecalis*	paused	
23	urology	prostate cancer	prostectomy	yes	3	MRI	joint + marrow + abscess	*Citrobacter koseri*	paused	
24	urology	prostate hypertrophy	TURP surgery	no	6	CT + MRI + PET/CT	bone + marrow + abscess + increased FDG	*Enterobacteria cloacae*	paused	
25	medicine	none	none	no	2	CT + MRI + PET/CT	joint + abscess + increased FDG	none	paused	
26	urology	tetraplegia	suprapubic catheter	no	2	CT + MRI	bone + marrow + abscess	*Escherichia coli*	yes	

“Referring department” refers to the department the patient was hospitalized before/after surgery and long-term follow-up was conducted. “Surgery” as in previous surgery in the pelvic area before SAS. “Radiotherapy” as in radiation therapy of the pelvic area. “Diagnostic delay” as in number of months with symptoms before SAS was diagnosed. “Imaging results” describes changes in bone (destruction/erosion), joint (swelling), marrow (edema) and soft tissue (abscess and fistula) in/near the symphysis. PET/CT and MRI description was missing in four cases. “Antibiotic” as in antibiotic treatment before SAS surgery. “Yes” means antibiotic treatment was not paused before surgery. “Yes ${}^{*}$ ” specifies the antibiotic treatment was not paused due to ongoing infection. “Paused” means antibiotic treatment was paused > 7 d before surgery. FDG – fluorodeoxyglucose. TURP – transurethral resection of the prostate.

### 3.2 Radiographic results

**Figure 2:**
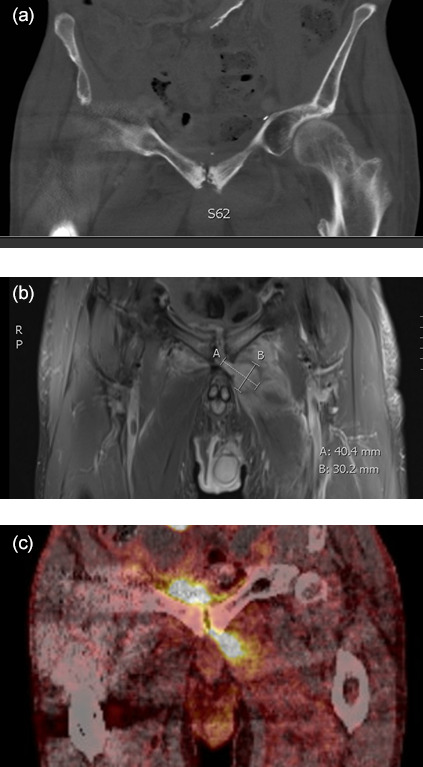
Example of three coronal diagnostic images before surgery from the same patient suggesting SAS diagnosis. **(a)** CT scan displaying osseous destruction in the symphysis. **(b)** MRI displaying an intramuscular abscess left to the symphysis. **(c)** PET/CT with increased FDG uptake in the symphysis and adjacent areas. The examinations were performed at the Department of Radiology and the Department of Nuclear Medicine and PET-Centre, Aarhus University Hospital, Denmark.

Patients were examined using combined imaging modalities (Fig. 2); the results are presented in Table 1. Magnetic resonance imaging (MRI) ( n = 24), computer tomography (CT) scan ( n = 17) and/or positron emission tomography (PET)/CT ( n = 10) imaging presented bone destructions/erosion in the pubis symphysis ( n = 14), bone marrow edema in the pubic rami ( n = 17), joint accumulation ( n = 16) and abscess around the symphysis ( n = 12). Five patients had a fistula communicating with the symphysis from the resected prostate area ( n = 2) and the bladder ( n = 3).

### 3.3 Microbiology results

The distribution of cultures is displayed in Table 1. *Staphylococcus aureus*, *Escherichia coli* and *Enterococcus faecalis* were the most common pathogens. Nine patients received intravenous or oral antibiotics at the time of surgery, but only one of the nine patients had a negative culture. In total eight cultures were negative even though intraoperative pus was present.

### 3.4 Treatment and outcome

Twenty-one patients were sufficiently treated for SAS after single-stage surgery and received at least 6 weeks of postoperative antibiotic treatment. Five patients had revision surgery, of which one patient underwent two revisions (Table 2).

**Table 2:** Details of the infection recurrence group.

	Predisposing	Surgery in	Radio-	Initial	Recurrent	Presentation	Months to	Intervention	Outcome	Complications
	conditions	the pelvic area	therapy	microbiology	microbiology	of recurrence	recurrence			
1	stress incontinence	mesh surgery	no	*Escherichia coli*	none ${}^{a}$	pain and swelling of the symphysis, CT displaying bladder perforation	1	surgical revision, reduction of dead-space with an abdominal muscle flap	pain relief, mobilized with walking aids	none
2	prostate hypertrophy	TURP surgery, penile implant	no	none	no data ${}^{b}$	infection in penile implant	3	debridement and removal of infectedimplant	well at follow-up	none
3	prostate cancer	none	yes	none	coryneform bacteria,coagulase-negative staphylococci	swelling along ilium, MRI with signs of infection in the symphysis	5	surgical revision	pain reduction, mobilized with walking aids	pelvic insufficiency fracture after radiotherapy, prolonged wound healing (5 months)
4	bladder cancer	cystectomy + Bricker bladder	yes	none ${}^{a}$	none ${}^{a}$	purulent surgicalwound drainage	1	surgical revision	pain reduction, mobilized with walking aids	pelvic insufficiency fracture after radiotherapy
5	prostate cancer	prostectomy and Brickerbladder	yes	*Streptococcus* (polyculture), *Escherichia coli*	non-hemolytic *Streptococcus*, *Escherichia* *coli*	pain and swelling of the symphysis and later recurrent sinus	0.5	surgical revision, debridement and excision of sinus after 6 months	pain reduction	prolonged wound healing, the patient is living with a chronic sinus.

${}^{a}$ Received continuous antibiotics before revision surgery due to current infection. ${}^{b}$ Information not present in patient record.

Four of the 26 surgical SAS debridements included the construction of a Bricker bladder by urologists, and one procedure included the mobilization of a vertical rectus abdominis musculocutaneous (VRAM) flap by plastic surgeons. Six patients (including three of the revised patients) were diagnosed with an insufficiency fracture in the pelvic ring (four patients had received radiotherapy). In comparison to their experiences before surgery, 23 patients reported reduced pelvic pain and analgetic use at 6 weeks follow-up. Nineteen patients were able to walk without walking aids while 7 patients (3 patients had insufficiency fractures) still had pain in the pelvic area and could walk using one or two crutches.

## 4 Discussion 

We report our experiences after surgically treating SAS in a series of 26 consecutive patients during a 12-year period.

Key findings are the following:

- Patients exposed to previous surgery in the pelvic area and presenting with persistent pubic/ suprapubic pain should be suspected of SAS.
- MRI is an essential tool to support the SAS diagnosis and in the preoperative planning.
- A multidisciplinary approach is recommended in the diagnostic, surgical and antibiotic management of SAS.
- Surgical treatment of SAS is a potential curative treatment leading to reduced pelvic pain and ambulation within 6 weeks.
- Our antibiotic regimen involved intraoperative topical gentamycin, 2 weeks of intravenous following 4 weeks of oral antibiotics.

### 4.1 Demographic

All patients in our cohort suffered from pubic or suprapubic pain as the primary symptom, which is coherent with the previous findings (Ross and Hu, 2003; Becker et al., 2020). This is usually associated with pain localized to the groin, thigh and hip, presumably because of radiating pain along the hip adductors. However, symptoms can be vague, fever can be absent, or the pain can be localized to only the hip or the abdomen, all contributing to the potential diagnostic delay, that in our cohort was up to 1 year. Twenty-one patients were exposed to previous surgery in the pelvic or abdominal region, of which 11 patients had prostate cancer. In a case series of 10 patients with SAS after previous prostate cancer surgery, considerable time from onset of symptoms to correct diagnosis was also reported (Gupta et al., 2015). Other risk factors should make clinicians consider the SAS diagnosis: pelvic malignancies, recent urinary incontinence surgery and intravenous drug use (Ross and Hu, 2003). In our experience the most challenging patient had an advanced infection, previous radiotherapy, received anticoagulant and/or presented with fistula/sinus. All these factors led to wound healing problems, infectious hematoma, potential bone destruction and a need for collaboration with other specialties.

### 4.2 Imaging

The patients in our cohort were examined with different imaging modalities, for the reason that most patients were referred from other departments and hospitals with limited imaging options and/or limited knowledge concerning SAS. If the image was suspicious of SAS, but only visualized on CT or PET/CT, an MRI was performed, as soft tissue changes are easily visualized early in the course of the disease (Alaya et al., 2017), whereas pelvic radiographs/CT are relatively insensitive as bone changes occur later in the disease process (Cardoso et al., 2017). Furthermore, MRI provides important information about the adjacent tissues to the symphysis and their associated pathologies like abscess and edema. In our experience PET/CT was useful to identify other infection foci as the accuracy for diagnosing deep infection/osteomyelitis is good (sensitivity range between 86 %–94 % and specificity range 76 %–97 %; Govaert et al., 2017), albeit the capability to differentiate specific structures such as fistulas and abscess is low compared to MRI. Twenty-four patients were MRI scanned, which became the method of choice in our institution to support the diagnosis and for preoperative planning.

### 4.3 Multidisciplinary approach

Most patients were referred to our department as SAS developed as a complication to other predisposing conditions and/or after failed antibiotic treatment. Furthermore, five patients had fistulas connecting to the symphysis from the bladder or scar tissue after prostectomy, necessitating a need for the involvement of urology specialists. Recently, Becker et al. (2020) found that fistulas to the pubic symphysis had a hazard ratio of 5.1 for treatment failure; however only one patient with fistula in our cohort had recurrence of infection and developed a chronic sinus tract. We do recommend a multidisciplinary approach with radiologist, microbiologist and relevant surgical specialties depending on the predisposing condition leading to SAS, as some patients require surgery in adjacent organs, e.g., bladder or flap surgery. This recommendation is in line with several recently published papers (Gupta et al., 2015; Dudareva et al., 2017; Becker et al., 2020; Shu et al., 2021), and the teamwork will most likely reduce morbidity and postoperative complications after SAS surgery

### 4.4 Surgical treatment

Twenty-one patients were successfully treated with pubic bone and joint debridement, draining the joint abscess leading to reduced pubic pain within days and improved ambulation within weeks. There are no long-term studies describing the patient outcomes after SAS surgery, however; our results are similar to the early postoperative results by Gupta et al. (2015) describing reduced pelvic pain after SAS surgery. Seven patients in our cohort still used crutches and had residual pubic pain during walking at 6 weeks follow-up, which was partly due to insufficiency fracture of the pelvic ring. Even after wide pubic resections, symphyseal instability has not been described after SAS debridement (Gupta et al., 2015; Shu et al., 2021). This is in line with our results and may partly be explained by a restricted mobilization regimen during the fibrous healing of the debrided area. Five patients in our cohort underwent at least one revision surgery. Two revised patients had radiotherapy treatment for cancer near the pelvis prior to SAS surgery and presented with severe wound healing problems, which is a well-known complication to irradiation (Micha et al., 2006; Dormand et al., 2005). Two cases may have been avoided: (1) if wound drainage had been applied to evacuate postoperative hematoma and (2) if an inflatable penile implant near the infected site had been removed. This emphasizes the importance of sufficient dead-space management and foreign body removal in orthopedic infection surgery (Metsemakers et al., 2020a). Shu et al. (2021) used vancomycin- and tobramycin-impregnated polymethylmethacrylate (PMMA) beads to reduce the dead space after SAS debridement (Shu et al., 2021), whereas we attended the dead space with a gentamicin collagen sponge, which delivered high local antibiotic concentrations (Thomassen et al., 2020) but shrinks shortly after leaving an antibiotic saturated dead space. We believe this highlights the significance of aggressive surgical debridement with removal of necrotic and infected bone (Cierny and di Pasquale, 2006) and the importance of applying adjuvant topical antibiotic agents when dealing with osteomyelitis.

### 4.5 Microbiology and antibiotics

We collected tissues samples from all patients, and *Staphylococcus aureus* and *Escherichia coli* were the most common pathogens, which is similar to the findings from previous papers (Dudareva et al., 2017; Ross and Hu, 2003; Becker et al., 2020). In our cohort, 18 patients presented with a positive culture, whereas 8 patients were culture negative; but since intraoperative pus was present, they were considered infected. This is in line with the confirmatory criteria for fracture-related infections, stating that fistulas, intraoperative pus and/or pathogens identified from at least two deep tissue specimens are confirmatory signs (Metsemakers et al., 2018). Our proportion of negative cultures (8 out of 26) from SAS were higher than the Dudareva et al. (2017) proportion of negative cultures (8 out of 61) from osteomyelitis in pelvic bones (Dudareva et al., 2017). This may be explained by the circumstance that we only collected a few representative samples and not five intraoperative samples as suggested in recent published papers (Metsemakers et al., 2020b; Dudareva et al., 2017).

Some patients with SAS respond well to long-term antibiotic treatment and should as such not be treated operatively; however, it is suggested that > 50 % require surgical debridement (Ross and Hu, 2003). In cases with acute SAS (symptoms < 1 month) (Andole et al., 2011; Cardoso et al., 2017; el Mezouar et al., 2014; Alaya et al., 2017), systemic antibiotic should be considered as bone vascularity may not have been compromised and biofilm formation may not yet have developed (Zimmerli and Sendi, 2017). All of our patients had a diagnostic delay between 1 and 12 months, in which some had received antibiotics at their local hospital or referring department without infection control; thus they were not considered acutely infected. Due to infected implants, fistulas, lack of infection control and/or radiographic signs of severe infection, all patients were surgical debrided.

Following surgical debridement of SAS, the ideal duration of antibiotic therapy is not well described, but most papers concerning SAS recommend between 6–12 weeks (Gupta et al., 2015; Shu et al., 2021; Becker et al., 2020). The antibiotic therapy for fracture-related infections recommends 6 weeks of antibiotics in consolidated fracture osteomyelitis (Depypere et al., 2020), whereas chronic long bone osteomyelitis receives 6 to 12 weeks of antibiotics based on the results of the final culture (Spellberg and Lipsky, 2012; McNally et al., 2016). The OVIVA trial compared 6 weeks of intravenous with oral antibiotics after surgery of complex bone and joint infections and demonstrated non-inferiority of oral antibiotics, evaluated as treatment failure within 1 year (Scarborough et al., 2019). We found that most patients in our cohort responded well with debridement, topical gentamycin following 2 weeks of intravenous and 4 weeks of oral antibiotics based on the culture sensitivity results (Depypere et al., 2020). If prolonged antibiotic treatment is necessary, we recommend that the antibiotic strategy is planned in collaboration with a microbiologist.

### 4.6 Limitations

Although this study cohort, to the best of our knowledge, is the largest population surgically treated for SAS yet described, the population remains small and heterogeneous.

The cohort is included over a decade, and the retrospective study design allowed for inevitably changes in surgical procedures following the experiences of outcome. In this context, it is noteworthy that the main recurrences of infection occurred among the first patients included. We presume it was partly due to the surgical learning curve and the gradual establishment of a multidisciplinary team understanding SAS.

The clinical follow-up time was short as most patients were referred from other departments; nevertheless, all patients were seen in the orthopedic outpatient clinic after a median of 6 weeks by the operating surgeon (Klaus Kjær Petersen).

## 5 Conclusions

SAS are rare conditions, occurring often after pelvic surgery and presenting with symptoms such as pelvic pain and impaired gait. The extent and severity of the infection can be visualized by MRI. Treatment of choice is early surgical debridement, often in collaboration with surgeons from other relevant departments, followed by at least a 6-week antibiotic regimen. The antibiotic regimen should always be planned in collaboration with a microbiology specialist.

For most patients this resulted in pain relief, improved walking ability and a low recurrence rate.

## Data Availability

Patient specific data cannot be provided without permission from the Danish Data Protection Agency.

## References

[bib1.bib1] Alaya Z, Zaghouani H, Osman W, Hassini L, Naouar N, ben Ayèche ML, Bouajina E (2017). Septic arthritis of the pubis symphysis: clinical and therapeutic features. Pan Afr Med J.

[bib1.bib2] Andole SN, Gupta S, Pelly M (2011). Septic arthritis affecting pubic symphysis. BMJ Case Rep.

[bib1.bib3] Becker A, Triffault-Fillit C, Valour F, Boussel L, Ruffion A, Laurent F, Senneville E, Chidiac C, Ferry T (2020). Pubic osteomyelitis: Epidemiology and factors associated with treatment failure. Med Mal Infect.

[bib1.bib4] Cardoso L, Alves P, Santos F, Ross JJ (2017). Septic arthritis of the pubic symphysis. BMJ Case Rep.

[bib1.bib5] Cierny G, di Pasquale D (2006). Treatment of chronic infection. J Am Acad Orthop Surg.

[bib1.bib6] Depypere M, Kuehl R, Metsemakers WJ, Senneville E, McNally MA, Obremskey WT, Zimmerli W, Atkins BL, Trampuz A (2020). Recommendations for Systemic Antimicrobial Therapy in Fracture-Related Infection: A Consensus From an International Expert Group. J Orthop Trauma.

[bib1.bib7] Dormand EL, Banwell PE, Goodacre TEE (2005). Radiotherapy and wound healing. Int Wound J.

[bib1.bib8] Dudareva M, Ferguson J, Riley N, Stubbs D, Atkins B, McNally M (2017). Osteomyelitis of the Pelvic Bones: A Multidisciplinary Approach to Treatment. J Bone Joint Infect.

[bib1.bib9] el Mezouar I, Abourazzak FZ, Mansouri S, Harzy T (2014). Septic arthritis of the pubic symphysis: a case report. Pan Afr Med J.

[bib1.bib10] Gomella P, Mufarrij P (2017). Osteitis pubis: A rare cause of suprapubic pain. Rev Urol.

[bib1.bib11] Govaert GA, Ipma FF, McNally M, McNally E, Reininga IH, Glaudemans AW (2017). Accuracy of diagnostic imaging modalities for peripheral post-traumatic osteomyelitis – a systematic review of the recent literature. Eur J Nucl Med Mol Imaging.

[bib1.bib12] Gupta S, Zura RD, Hendershot EF, Peterson AC (2015). Pubic symphysis osteomyelitis in the prostate cancer survivor: clinical presentation, evaluation, and management. Urology.

[bib1.bib13] McNally MA, Ferguson JY, Lau ACK, Diefenbeck M, Scarborough M, Ramsden AJ, Atkins BL (2016). Single-stage treatment of chronic osteomyelitis with a new absorbable, gentamicin-loaded, calcium sulphate/hydroxyapatite biocomposite: a prospective series of 100 cases. Bone Joint J.

[bib1.bib14] Metsemakers WJ, Morgenstern M, McNally MA, Moriarty TF, McFadyen I, Scarborough M, Athanasou NA, Ochsner PE, Kuehl R, Raschke M, Borens O, Xie Z, Velkes S, Hungerer S, Kates SL, Zalavras C, Giannoudis Pv, Richards RG, Verhofstad MHJ (2018). Fracture-related infection: A consensus on definition from an international expert group. Injury.

[bib1.bib15] Metsemakers WJ, Fragomen AT, Moriarty TF, Morgenstern M, Egol KA, Zalavras C, Obremskey WT, Raschke M, McNally MA (2020). Evidence-Based Recommendations for Local Antimicrobial Strategies and Dead Space Management in Fracture-Related Infection. J Orthop Trauma.

[bib1.bib16] Metsemakers W-J, Morgenstern M, Senneville E, Borens O, Govaert GAM, Onsea J, Depypere M, Richards RG, Trampuz A, Verhofstad MHJ, Kates SL, Raschke M, McNally MA, Obremskey WT, group O behalf of the F-RI (FRI) (2020). General treatment principles for fracture-related infection: recommendations from an international expert group. Arch Orthop Trauma Surg.

[bib1.bib17] Micha JP, Goldstein BH, Rettenmaier MA, Caillouette JT, Fee MJ, Brown 3rd JV (2006). Pelvic radiation necrosis and osteomyelitis following chemoradiation for advanced stage vulvar and cervical carcinoma. Gynecol Oncol.

[bib1.bib18] Ross JJ, Hu LT (2003). Septic arthritis of the pubic symphysis: review of 100 cases. Medicine (Baltimore).

[bib1.bib19] Scarborough M, Li HK, Rombach I, Zambellas R, Walker AS, McNally M, Atkins B, Kümin M, Lipsky BA, Hughes H, Bose D, Warren S, Mack D, Folb J, Moore E, Jenkins N, Hopkins S, Seaton RA, Hemsley C, Sandoe J, Aggarwal I, Ellis S, Sutherland R, Geue C, McMeekin N, Scarborough C, Paul J, Cooke G, Bostock J, Khatamzas E, Wong N, Brent A, Lomas J, Matthews P, Wangrangsimakul T, Gundle R, Rogers M, Taylor A, Thwaites GE, Bejon P (2019). Oral versus intravenous antibiotics for bone and joint infections: the OVIVA non-inferiority RCT. Health Technol Assess.

[bib1.bib20] Shu HT, Elhessy AH, Conway JD, Burnett AL, Shafiq B (2021). Orthopedic management of pubic symphysis osteomyelitis: a case series. J Bone Joint Infect.

[bib1.bib21] Spellberg B, Lipsky BA (2012). Systemic antibiotic therapy for chronic osteomyelitis in adults. Clin Infect Dis.

[bib1.bib22] Thomassen MB, Hanberg P, Stilling M, Petersen KK, Søballe K, Krag LB, Højskov CS, Bue M (2020). Local concentrations of gentamicin obtained by microdialysis after a controlled application of a GentaColl sponge in a porcine model. J Orthop Res.

[bib1.bib23] Zimmerli W, Sendi P (2017). Orthopaedic biofilm infections. APMIS.

